# Orthodontic treatment of traumatically avulsed maxillary central incisors with bimaxillary dentoalveolar protrusion in an adult female: a case report

**DOI:** 10.1186/s12903-023-03123-7

**Published:** 2023-07-08

**Authors:** Xiaotong Yu, Xiaoni Duan, Cheng Zhi, Yilin Jiang, Ziyi Chen, Chunxiang Zhang

**Affiliations:** 1 Department of Orthodontics, Tianjin Stomatological Hospital, School of Medicine Nankai University, Tianjin, 300041 China; 2 Tianjin Key Laboratory of Oral and Maxillofacial Fuction Reconstruction, Tianjin Stomatological Hospital, Tianjin, 300041 China; 3 Department of Orthodontics, Stomatology Hospital, School of Stomatology, Zhejiang University School of Medicine, Hangzhou, 310006 China; 4 School and Hospital of Stomatology, Tianjin Medical University, Tianjin, 300070 China; 5 School and Hospital of Stomatology, China Medical University, Shenyang, 110001 China

**Keywords:** Incisor missing, Trauma, Space closure, Multidisciplinary

## Abstract

**Background:**

Clinicians agree that obtaining and retaining good treatment results for missing maxillary central incisors owing to trauma is not easy. Management of adult patients with permanent maxillary central incisor loss who visit the clinic with high expectations for aesthetics and function pose a significant diagnostic dilemma. Therefore, esthetic and functional outcomes should be taken into consideration when deciding the proper treatment method. The treatment described in this study aimed to reestablish smile esthetics by proposing an effective multidisciplinary clinical approach that includes orthodontic-prosthetic-periodontal procedures, optimally reduced lip protrusion, center dental midlines, and establishment of stable occlusion.

**Case presentation:**

The patient was a 19-year-old adult female with bimaxillary arch protrusion who had been wearing removable dentures for several years since the loss of her maxillary central permanent incisors. A multidisciplinary treatment including the extraction of two mandibular primary premolars was adopted. The treatment plan consisted of orthodontic space closure by shifting the adjacent teeth towards the central incisor spaces combined with appropriate morphologic remodeling and gingival reshaping to obtain good aesthetic and functional results. The duration to complete the orthodontic treatment was 35 months. Clinical and radiographic results after treatment suggested smile harmony with an improvement in the facial profile, good function of the occlusion, and a positive effect on bone remodeling in the area of the missing incisors during orthodontic tooth movement.

**Conclusions:**

This clinical case illustrated the necessity for using multidisciplinary methods involving orthodontic, prosthodontic, and periodontic procedures to treat an adult female patient with bimaxillary arch protrusion and long-term absence of anterior teeth due to severe trauma.

## Background

Dental trauma is the second most critical disease following carious lesions causing permanent dentition defects in children. They are defined as acute damage to the hard tissues, pulp, or periodontium of the tooth by the action of sudden external forces [1]. According to various studies, the incidence of dental injuries among adolescents ranges from 4 to 35% [2–5]. A 2011 study reported the highest occurrences between ages 7 and 10, and dental trauma occurs more often in adolescent boys than in girls [4]. The maxillary central incisors are most commonly affected (84%), followed by the mandibular central incisors (7.5%), maxillary lateral incisors (4.5%), and maxillary canines (3%) [6]. The long-term absence of anterior teeth can cause anterior diastema and the tipping of the adjacent teeth, which will affect masticatory and phonic functions as well as the growth and development of the jaws, thus aggravating the difficulty of treatment.

There are several options for malocclusion in patients with long-term loss of anterior teeth: removable dentures, fixed prosthetic restorations, implant prosthetic restorations, and treatment combined with aesthetic restorations after orthodontic autonomous closing of the avulsed space by the migration of adjacent teeth. However, the patients presenting with traumatic tooth loss or aesthetic complaints are mostly young adults with high expectations for aesthetics and function, who are increasingly paying attention to the harmonious relationship between teeth and face. Treatment with orthodontic or restorative modality alone is often not ideal [7]. The one-sided orthodontic approach, which just autonomously closes the avulsed space by the migration of adjacent teeth, fails to obtain proper coordination of the upper and lower teeth, and barely meet the future esthetic and functional needs. For adult patients with malocclusion, if the restorative treatment is performed directly to eliminate the missing tooth space without getting proper orthodontic treatment, undesirable conditions such as difficulty in obtaining a common path of insertion for fixed prostheses, increased abutment damage, easy fracturing of the denture, and difficulty in establishing a stable bite may occur. Orthodontic, prosthodontic, and periodontic treatment combined can effectively control root movement of the adjacent teeth, centralize the allocation of the missing tooth space, adjust proper axial inclination of the teeth, minimize the damage to the abutment teeth, and facilitate dental function and periodontal health. In addition, the patient's articulation and chewing function are further protected. Therefore, a comprehensive treatment plan should be designed. First, the position of the remaining teeth should be reasonably adjusted through orthodontic treatment, then the prosthodontic and periodontic treatments should be selected to obtain the best esthetic results.

This case report discusses an interdisciplinary method in which the lateral incisors were moved mesially to substitute for central incisors by orthodontists, reshaped to resemble central incisors by prosthodontist, and an optimum level for the marginal gingival contours was achieved by periodontists.

## Case presentation

### Diagnosis and etiology

A 19-year-old adult female patient sought orthodontic treatment with the chief complaint of the loss of the upper anterior teeth and a convex surface type. Clinical examination was performed, and orthodontic records were obtained, including lateral skull radiographs and cone-beam computed tomography (CBCT). The patient was healthy and had been wearing removable dentures for several years since the loss of the incisors. She had no history of systemic disease or harmful oral habits, and no contraindications to orthodontic treatment or periodontal problems.

**Fig. 1:**
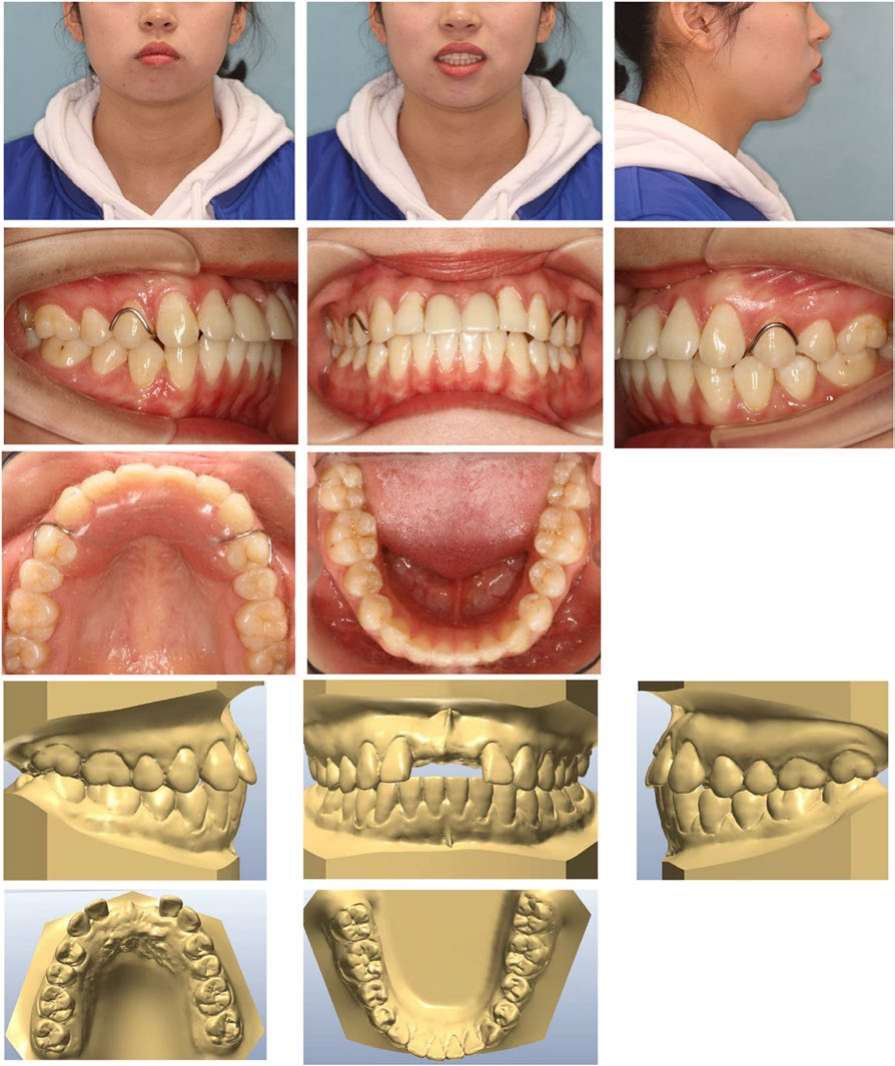
Pretreatment facial and intraoral photographs and dental casts

**Fig. 2:**
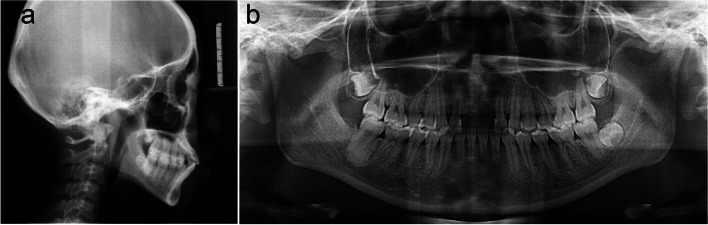
**a** Pretreatment radiographs: lateral cephalogram. **b** panoramic radiograph

On examination, she had a normal temporomandibular joint. The pretreatment facial analysis demonstrated an acceptable frontal view, facial symmetry, and balanced facial thirds, adequate smile characteristics, and upper incisors that were not visible on display at rest (Fig. 1). The side view suggested a convex soft-tissue profile with a smaller nasolabial angle of 81.6° (Fig. 1). Intra-oral and dental cast examinations showed a Class I molar and canine relationship, missing maxillary central incisors (Fig. 1). The cephalometric analysis confirmed a Class I skeletal relationship with protrusive maxillary and mandible incisors and a vertical growth pattern (Fig. 2a). According to the panoramic radiograph, although bone resorption was more obvious in the alveolar bone without roots, it could meet the need for orthodontic tooth movement to close the missing space (Fig. 2b).

### Treatment alternatives

Two treatment alternatives were offered to the patient and we addressed the risks and benefits. The first option, without extraction, suggested reserving the space of the missing maxillary central incisors for implantation/prosthodontic treatment. This option posed a shorter treatment course and was more predictable but had a limited impact on the facial appearance. The second option combined orthodontic, prosthodontic, and periodontic treatments with extraction of two mandibular first premolars, including retracting anterior teeth, which would be controlled by self-tapping, screw-type, micro-implants to close the extraction space and, the missing central incisors would be replaced by the mesial movement of the lateral incisors. This would be followed by aesthetic and functional restoration of the maxillary lateral incisors, cuspids, and first premolars. This option would lead to a sustainable and non-detrimental improvement in appearance after treatment. Nevertheless, the treatment period is long, and potential root resorption or alveolar bone loss, including fenestration and dehiscence due to insufficient bone volume in the edentulous area during treatment, could occur. Therefore, bone grafting could not be ruled out. The second treatment option was adopted. However, only lateral incisors were reshaped, the canines and premolars were not.

### Treatment progress

**Fig. 3:**
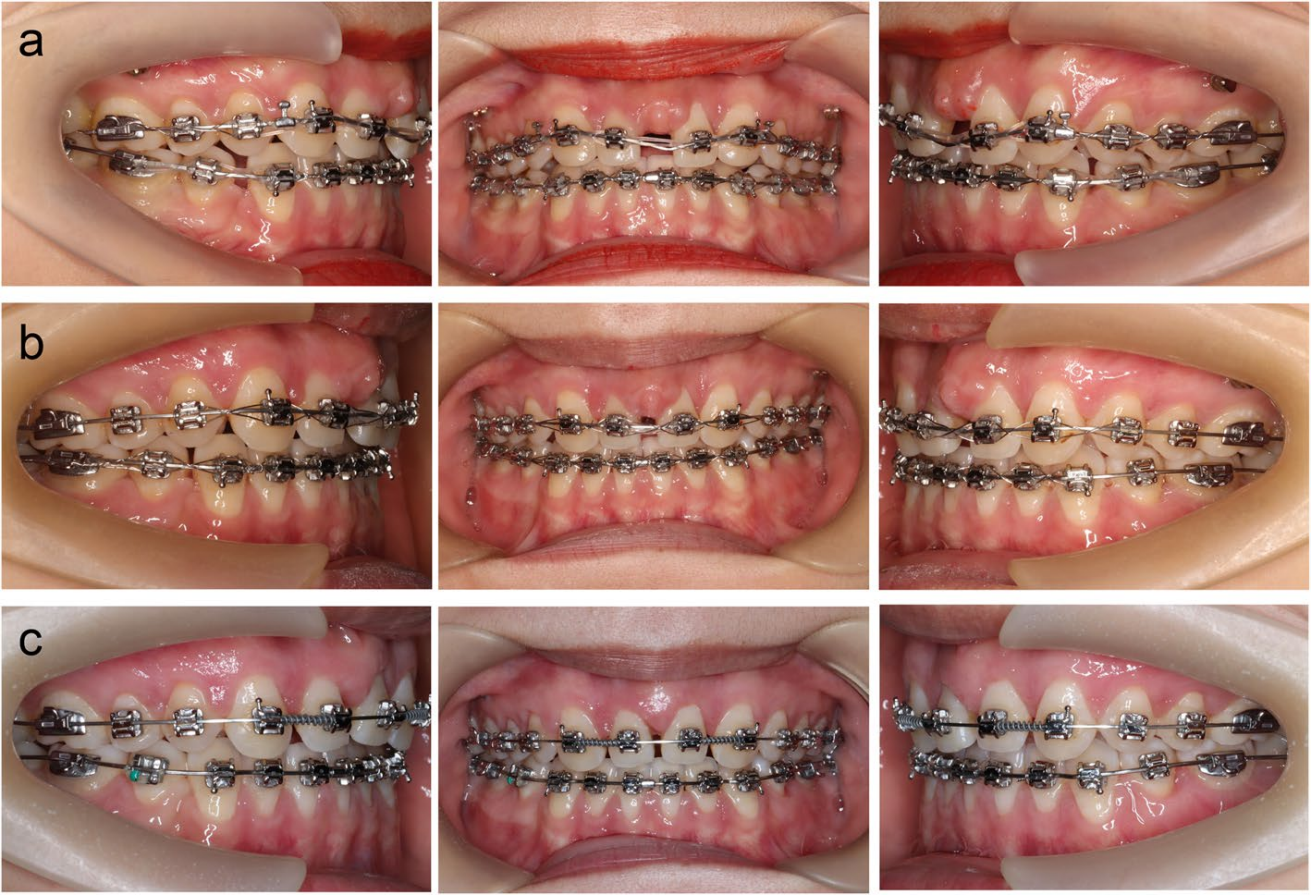
Progress intraoral photographs. **a** Control torque with 018 × .0.25 inches stainless steel wire after 17 months of treatment. **b** Close space with .018 × .0.25 inches stainless steel wire by elastics chain wire after 25 months of treatment. **c** One month after gingivectomy and gingivoplasty

The removal of the mandibular first premolars was performed by oral surgeons. One month after the micro-implants (8 mm long, 1.2 mm in diameter) were placed into the interradicular bone between the maxillary second premolar and first molar on both sides, preferably between the attached and movable mucosae, a preadjusted fixed appliance, 0.022 × 0.025 inches was placed in both arches. Maxillary central incisor brackets were placed on the lateral incisors to allow a more palatal root torque and reduce the mesial inclination. Initially, the teeth were leveled with a sequence of .012, .014, .016 inches nickel-titanium wire for 4 months, and only the maxillary canines were passively ligated with micro-implants to avoid unwanted mesial displacement during this phase. Medical metal wire (Φ 0.20 mm) ensured active ligation between the maxillary lateral incisors where the second-order bend was finished for further torque control sequence. This was switched from .016  ×  .0.22 inches rectangular nickel-titanium wire to .018  ×  .025 inches stainless steel wire when the space was beginning to close with elastic chain wiring in both arches (Fig. 3a and b). Before removing the brackets, the patient was referred to the periodontal department for gingivectomy and gingivoplasty to improve her gingival margins (Fig. 3c).

**Fig. 4:**
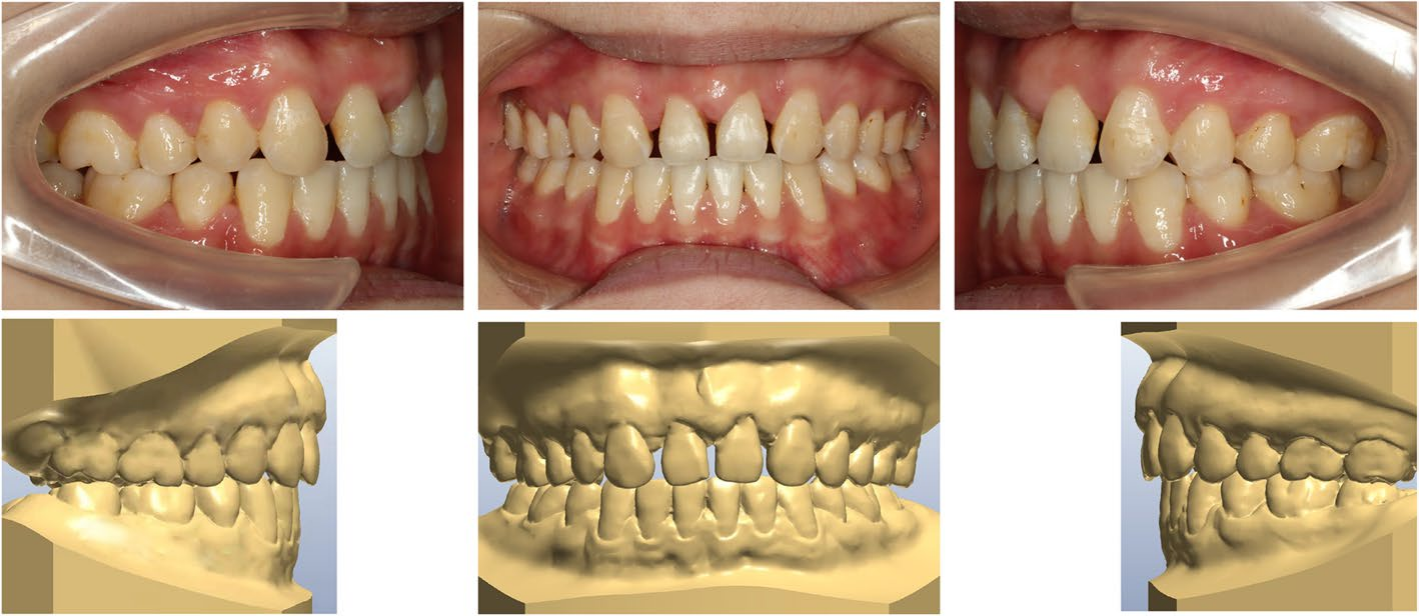
Intraoral photographs and dental casts before prosthetic rehabilitation of maxillary lateral incisor

The duration to complete the orthodontic treatment was 35 months, which was substantially extended due to missed appointments resulting from the coronavirus disease pandemic and broken appliances. The interdisciplinary discussion had been ongoing, especially in the later stage of treatment. The appliance was removed when the final position of the lateral incisors, both esthetically and functionally, was agreed upon. Subsequently, the patient was referred to the prosthodontists (Fig. 4). After the dental esthetic treatment, the patient's retainer was remade, and the patient was instructed to wear the retainer strictly according to the doctor's advice for at least two years and follow the prescribed period of time for follow-up visits.

**Fig. 5:**
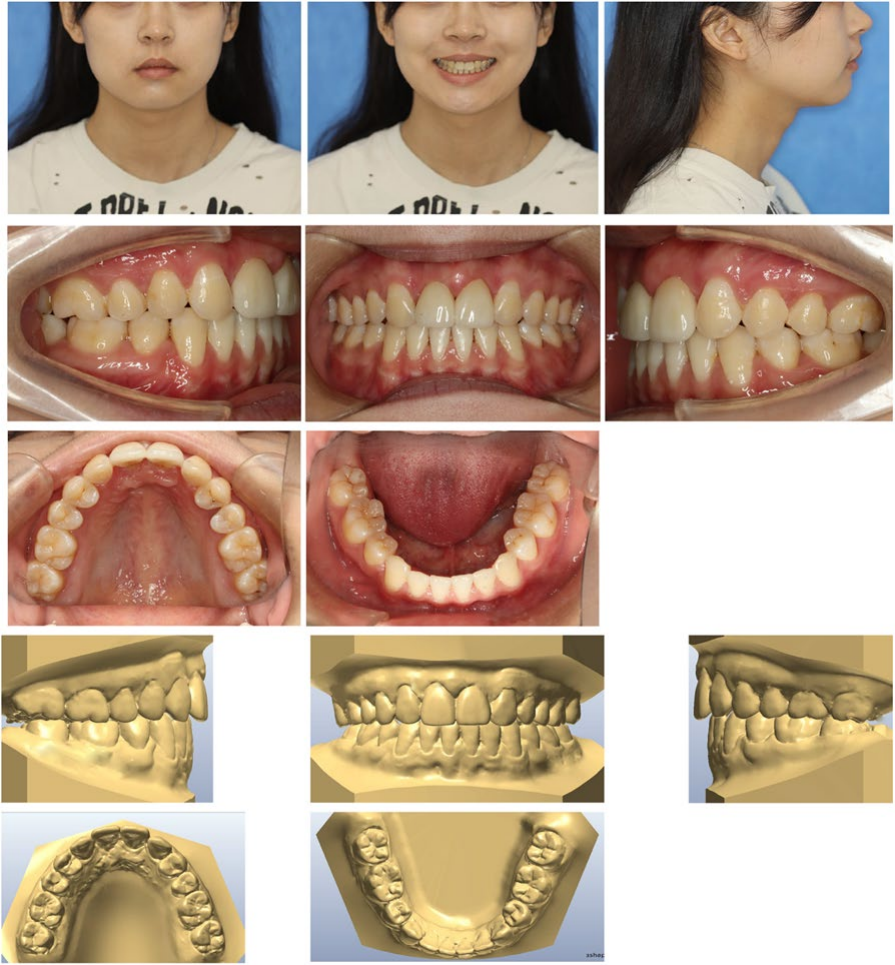
Posttreatment facial and intraoral photographs and dental casts

**Fig. 6:**
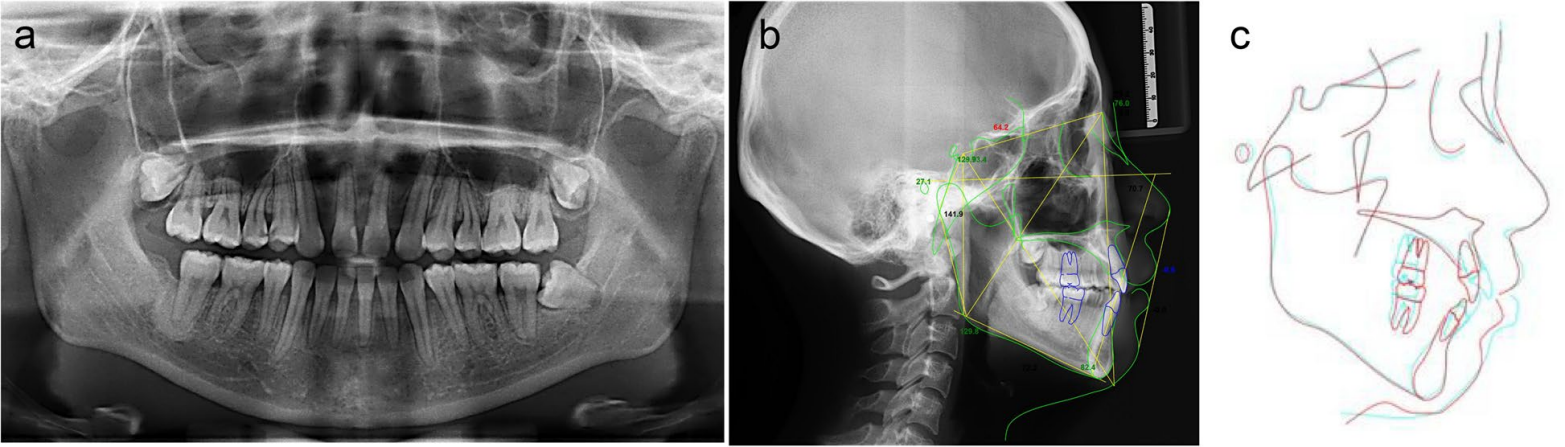
Posttreatment radiographs. **a** Panoramic radiograph. **b** Lateral cephalogram. **c** Cephalometric superimposition of pretreatment and posttreatment

**Fig. 7:**
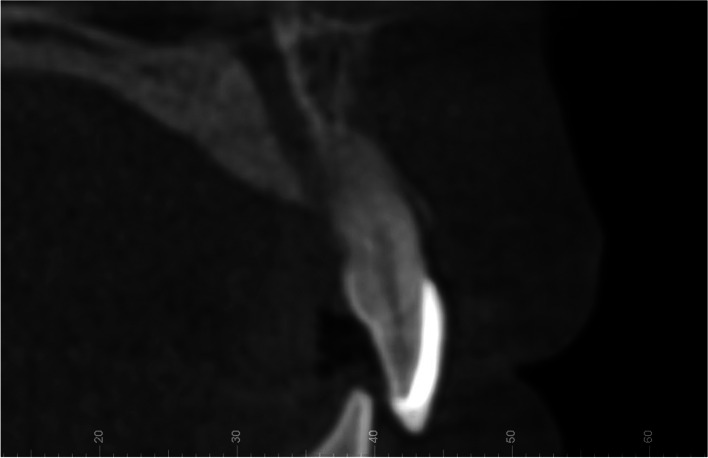
Bone remodeling of maxillary central incisors after treatment

The final extraoral photographs showed a harmonious smile and good facial aesthetics with significant improvement in the facial profile. The edentulous spaces were closed and occupied by adjacent teeth, along with a satisfactory overbite and overjet. The patient had been advised to have her wisdom teeth removed as soon as possible after returning to their place of residence. The only regret was that the midlines of the upper and lower teeth did not exactly coincide, and due to the asymmetrical extractions, interproximal enamel reduction was suggested during treatment to harmonize the Bolton ratio. However, the patient refused and accepted the midline inconsistency (Fig. 5). The patient ended up removing the aligners in a hurry due to graduation. Posttreatment panoramic radiography showed unperfected root parallelism without any appreciable bone loss or root resorption (Fig. 6a). Superimposed cephalometric tracings (initial, final) (Fig. 6a and b), as well as their numeric values (Table 1), indicated the improvement in the angle formed by point N (nasion), point A (subspinale), and point B (supramental), mainly caused by bone remodeling of the anterior maxilla due to lateral incisor movement (Fig. 7).

**Table 1:** Cephalometric analysis at pretreatment and posttreatment

**Measurements**	**Norm (mean ± SD)**	**Pretreatment**	**Posttreatment**
SNA (°)	82.8 ± 4.0	77.2	79.2
SNB (°)	80.1 ± 3.9	75.9	76.0
ANB (°)	2.7 ± 2.0	1.3	3.2
NP-FH (°)	85.4 ± 3.7	85	85.3
NA-PA (°)	6.0 ± 4.4	2.2	5.7
UI-NA (mm)	5.1 ± 2.4	13.5	3.4
UI-NA (°)	22.8 ± 5.7	40	11.5
LI-NB (mm)	6.7 ± 2.1	10	4.3
LI-NB (°)	30.3 ± 5.8	31	18.2
UI-LI (°)	125.4 ± 7.9	109	147.1
UI-SN (°)	105.7 ± 6.3	112.5	90.6
MP-SN (°)	32.5 ± 5.2	46	39.3
FH-MP (°)	31.1 ± 5.6	33	30.4
LI-MP (°)	92.6 ± 7.0	96.7	88.2
YAix (°)	66.3 ± 7.1	64	64.8
Po-NB (°)	1.0 ± 1.5	1	1.6

## Discussion and conclusions

It is not easy for a clinician to create and retain good aesthetic and functional results for traumatically avulsed maxillary incisors. Major factors, such as the location and number of missing teeth, age, profile, the arrangement of teeth, and the general need for orthodontic treatment, should be considered after a thorough examination to determine the appropriate treatment. In this case, the patient chose orthodontic space closure by the migration of adjacent teeth, but the long-term loss of the maxillary central incisor resulted in alveolar ridge resorption, which adversely affected gap closure [8]. According to the basic orthodontic axiom of “bone tracking tooth movement,” the bone around the alveolar socket will remodel to the same extent when the orthodontic tooth moves [9]. When the assessment of the alveolar bone mass is acceptable, the orthodontist may consider the characteristics of the physiological response of the adjacent teeth and apply continuous light force to adaptively remodel the alveolar bone [10]. If the force is applied improperly, especially in adults with a relatively long course of treatment, the alveolar bone will be further absorbed, and an iatrogenic injury, such as alveolar bone dehiscence and fenestration, can occur [11]. The increased angle between the sella, nasion and subspinale point values measured by lateral cephalometric films after orthodontic treatment showed a good bone remodeling and increasing thickness of labial alveolar bone during the movement, which was important for the stability of the lateral incisors. For patients with severe alveolar bone defects, guided bone regeneration should be performed before orthodontic treatment to reduce the risk of tooth movement [12]. However, orthodontic space closure may not be appropriate in many cases. The following conditions, including convex shape, normal number of maxillary teeth with crowded dentition, deep overjet, or steep Spee curve, are suitable for this method. When the neutral relationship of the posterior teeth is established, the proportion of reconstructed anterior teeth should be emphasized, and the Bolton ratio of anterior teeth used to determine whether the mandibular teeth need to be carried out with interproximal enamel reduction.

To ensure a perfect gingival contour, excessive proximal inclination of the upper lateral incisors should be avoided during space closure [13]. In this case, the central incisor brackets were placed on the lateral incisors to reduce the mesial angulation while allowing for increased torque control of the root and selective use of second-order archwire adjustments to promote mesial movement of the lateral incisor root. The whole course was treated with light orthodontic force, and the space was closed slowly to achieve ideal root positioning. As the lateral incisors moved mesially, the canines also moved mesially. Prosthodontists have long objected to the mesial movement of the maxillary canines into the lateral incisor space because it ruled out the possibility of canine protection. However, Silveira et al. [14] justified the mesial movement of canines into lateral incisor space, which provided a theoretical basis for many orthodontists to close the space. They also pointed out that prosthetic replacement was worse with respect to periodontal indexes than orthodontic space closure treatment [14]. In addition, studies have shown there is no significant relationship between the presence or absence of cuspid protected occlusion and the incidence of temporomandibular joint dysfunction [15, 16]. This leads to the assumption that the Class I relationship of the canines is not a necessary prerequisite for the presence of a canine-protected occlusion.

The emergence profile of the maxillary central incisor on the mesial surface was generally flat; therefore, the lateral incisors were approximated to allow for an optimal profile of the restorations. Subsequent application of porcelain veneers can contribute to a harmonious smile and patient satisfaction [17]. Although the patient required some adjunctive procedures to the bicuspid and first premolars for complex plastic surgery, namely, preventing interferences by grinding the palatal cusp of the first premolar and bleaching where the colors did not match, the patient denied it as she was satisfied with the results.

In the process of the maxillary lateral incisors mesial movement, the speed of gingival remodeling is slower than that of the teeth, which results in the gingival accumulation and abnormal marginal morphology in the central region of the maxilla [18]. Gingiva accumulation may occur between the adjacent teeth for larger spaces, especially if the orthodontic process is too fast [19, 20]. This has alarming effects on clinical work. Therefore, the correction speed should not be fast; the obvious gingival accumulation has a negative effect on aesthetics and retaining the orthodontic result. Therefore, before removing the brackets, the patient was referred for gingivectomy and gingivoplasty to improve her gingival margins.

It is important to remember the patient in this case complained of the need to improve the facial shape to a large extent. Therefore, in this experimental design process, based on ensuring a sufficient amount of apical bone, the closure of the edentulous gap was completed by moving the anterior teeth inward. The overall support of the micro-screws in the pre-correction period led to insufficient control of the anterior torque. But what I need to reflect on was the insufficient control of anterior tooth torque during gap closure process, which resulted in the loss of anterior tooth torque. In future treatments, I will be reminded even more of the necessity of controlling anterior tooth torque.

The necessity for using multidisciplinary methods to treat traumatic loss of anterior teeth has been emphasized for many years. This case report presents a treatment program that carefully evaluated the facial features, dentition, and bone structure, followed by gingivoplasty, appropriate remodeling, and veneering after orthodontic space closure. Thus, successful interdisciplinary management of a condition critically relies on patient compliance and cooperation and good team performance throughout the treatment process.

## Abbreviation

CBCT: Cone-beam computed tomography
